# 

**Published:** 1925

**Authors:** 


*Editor:* PATRICK WATSON-WILLIAMS, M.D.,
WITH WHOM ARE ASSOCIATED
J. LACY FIRTH, M.S., M.D., CAREY COOMBS, M.D.,
E. W. HEY GROVES, M.S., F.R.C.S., B.Sc.
J. A. NIXON, C.M.G., M.D., *Assistant Editor.*
*Editorial Secretary:* A. L. FLEMMING, M.B., B.Ch.

Local Medical Notes ... ... ... ... ... ... ... ... 154

BRISTOL: J. W. ARROWSMITH LTD., 11 QUAY STREET.
LONDON: J. W. ARROWSMITH (LONDON) LTD., 6 UPPER BEDFORD PLACE, RUSSELL SQUARE.

### The Library of the Bristol Medico-Chirurgical Society.

*The following publications have been received since the publication of the List in January, 1925.*

*May*, 1925.

| | | |
|---|---|---|
| American Laryngological Society (1) | .. .. | 1 volume. |
| Australasian Medical Congress (2) | .. .. | 1 ,, |
| Mr. F. Richardson Cross (3) | .. .. .. | 1 ,, |
| Dr. D. S. Davies (4) | .. .. .. .. | 1 ,, |
| Delegates of the Clarendon Press (5) | .. .. | 2 ,, |
| Dr. J. H. Drysdale (13) | .. .. .. .. | 19 ,, |
| Mr. E. W. Hey Groves (6) | .. .. .. | 1 ,, |
| Dr. David Hooper (7) | .. .. .. .. | 2 ,, |
| Munro-Smith Fund (8) | .. .. .. .. | 3 ,, |
| Radium Institute (9) | .. .. .. .. | 1 ,, |
| Smithsonian Institution (10) | .. .. .. | 1 ,, |
| Mr. A. Rendle Short (11) | .. .. .. | 1 ,, |
| Dr. P. Watson-Williams (12) | .. .. .. | 1 ,, |

Unbound periodicals have been received from Dr. Carey Coombs, Mr. Flemming, Mr. Hey Groves, Dr. J. A. Nixon, and Dr. B. M. H. Rogers.

### THE ONE HUNDRED AND TWELFTH LIST OF BOOKS.

The figures in round brackets refer to the figures after the names of the donors. The books to which no such figures are attached have either been bought from the Library Fund or received through the *Journal*.

| | | |
|---|---|---|
| **Barton, E. A.** .. | *Essentials of Infant Feeding* .. .. .. .. | 1925 |
| **Beaumont and Dodds** | *Recent Advances in Medicine* .. .. .. (11) | 1925 |
| **Berkeley, Comyns** | *Gynæcology for Nurses* .. .. .. 4th Ed. | 1925 |
| **Bertwistle, A. P.** .. | *Atlas of Radiographs of the Bones and Joints* | 1924 |
| **Bourne, A. W.** .. | *Synopsis of Midwifery and Gynæcology* 3rd Ed. | 1925 |
| **Cameron, S. J.** .. | *A Manual of Gynæcology* .. .. 3rd Ed. | 1925 |
| **Carson, H. W.** [Ed.] | *Modern Operative Surgery.* 2 vols. .. .. | 1924 |
| **Central Midwives Board.** | *Examination Questions and Model Answers* .. .. .. .. .. .. .. .. 2nd Ed. | 1925 |
| **Chesser, Eliz. S.** [Ed.] | *Health and Psychology of the Child* .. .. | 1925 |
| **Chetwood, C. H.** .. | *The Practice of Urology* .. .. .. .. .. | 1925 |
| **Chiene, G. L.** .. | *Handbook of Surgery* .. .. .. .. .. (6) | 1923 |

**Cope, Z.** .. .. .. *Clinical Researches in Acute Abdominal Disease* .. .. .. .. .. .. .. .. .. 1925
**Cross, F. R.** .. .. *The Brain Structures concerned in Vision* (3) [1909]
**Cumberbatch and Robinson.** *Treatment of Gonococcal Infection by Diathermy* .. .. .. .. .. .. .. .. .. 1925
**Cushing, H** .. .. *Sir William Osler.* 2 vols. .. .. .. (5) 1925
**Dukes, C.** .. .. *The Bacteriology of Food* .. .. .. .. .. .. 1925
**Falta, W.** .. .. *Endocrine Diseases* .. .. .. .. .. .. .. 1924
**Groves, E. W. Hey** *Surgical Operations* .. .. .. .. 2nd Ed. 1925
**Groves and Brickdale.** *Textbook for Nurses* .. .. .. .. 3rd Ed. 1925
**Henry, T. A.** .. *The Plant Alkaloids* .. .. .. .. .. .. .. 1925
**Hyslop, T. B.** .. *The Borderland* .. .. .. .. .. .. .. .. 1924
**Jones, F. W.** .. .. *The Principles of Anatomy as seen in the Hand* 1920
**Jones, W. H. S.** .. *The Doctor's Oath* .. .. .. .. .. .. (8) 1924
**Kaye, G. W. C.** .. *X-Rays* .. .. .. .. .. .. 4th Ed. (8) 1923
**Kennedy, A. M.** .. *Parasitology for Medical Students* .. .. .. 1925
**Kolmer, J. A.** .. *Infection, Immunity and Biologic Therapy* 3rd Ed. 1924
**Laird, J.** .. .. .. *Tuberculosis: Its Prevention and Treatment* 2nd Ed. 1925
**Marlow, F. W.** .. *The Relative Position of Rest of the Eyes* .. (4) 1924
*Medical Annual, The,* 1925 .. .. .. .. .. .. .. .. .. .. 1925
**Pinch, A. E. H.** .. *A Clinical Index of Radium Therapy* .. (9) 1925
**Rogers, Sir L., and Muir, E.** *Leprosy* .. .. .. .. .. .. .. .. 1925
**Savage, W. G.** .. *Canned Food in relation to Health* .. (12) 1923
**Sheard, A.** .. .. *Pernicious Anæmia and Aplastic Anæmia* .. 1924
**Singer, C., and Singerist** [Ed.]. *Essays on the History of Medicine* (8) 1924
**Tweedy and Others.** *Practical Obstetrics* .. .. .. .. 5th Ed. 1925
**Walker, Sir N.** .. *An Introduction to Dermatology* .. 8th Ed. 1925
**Wesselow, O. L. V. de.** *The Chemistry of the Blood in Clinical Medicine* .. .. .. .. .. .. .. .. .. 1924
**Wigg, H.** .. .. *Medical Lectures for Nurses* .. .. .. .. .. 1915
**Williams, L.** .. .. *Middle Age and Old Age* .. .. .. .. .. .. 1925
**Weaver, G. H.** [Ed.] **and Others.** *The Practical Medicine Series,* Vol. I. .. .. .. .. .. .. .. .. .. .. .. 1924

### TRANSACTIONS, REPORTS, JOURNALS, &c.

American Pharmaceutical Association Year Book, 1920 and 1922 (7) 1922-23
Annual Report of the Smithsonian Institution, 1922 .. .. (10) 1924
Australasian Medical Congress, Transactions of the First Session, 1923 .. .. .. .. .. .. .. .. .. .. .. .. .. (2) 1924
Medical Society Transactions .. .. Vols. XXVII. to XLV. (13) —
Surgical Clinics of North America, October, 1924 .. .. .. .. .. 1924
Transactions of the Ophthalmological Society of the United Kingdom. Vol. XLIV. .. .. .. .. .. .. .. .. .. .. .. .. .. 1924
Transactions of the Thirtieth Meeting of the American Laryngological Society .. .. .. .. .. .. .. .. .. .. .. .. (1) 1924

### Local Medical Notes.

**University of Bristol.** June 9th. Opening of the new buildings by H.M. The King.

Their Majesties The King and Queen will arrive at the University at 2.45 p.m. and will leave about 4 o'clock. Guests must be in their places in the Great Hall by 1.30 p.m.

After the departure of Their Majesties a number of guests have been invited by the Council and Senate to tea in the grounds of the Royal Fort House, and at 8.30 p.m. to a reception to be held in the University.

Founder's Day, June 12th. At 11 a.m. a special service will be held in the Cathedral. Preacher: The Right Rev. The Lord Bishop of Truro. At 3 p.m. the cricket match, Academic Staff *v.* The Undergraduates, will be played on the University Athletic Ground at Coombe Dingle.

**Opening of the new Homœopathic Hospital.**—On May 26th the new buildings of the Homœopathic Hospital were opened by Her Highness Princess Helena Victoria. The Hospital has been erected on the grounds of Cotham House, and is the gift of Mr. Melville Wills in memory of Captain Bruce Wills, his eldest son, who was killed in the War at St. Eloi while helping to carry a wounded officer. The building is a particularly handsome edifice, with modern and efficient equipment, including a complete installation of X-ray apparatus, the gift of Mr. Melville Wills.

**British Medical Association.**—At the Annual Meeting at Bath, July 21st to 24th, F. G. Thomson, M.A., M.D., M.R.C.P., will be elected President.

Many members of the Medico-Chirurgical Society and other colleagues of the Bath and Bristol Branch of the Association hold office in the various sections, viz.:—

*Medicine:* Vice-President, Professor J. A. Nixon, C.M.G., M.D., F.R.C.P.; Hon. Secretary, James Lindsay, M.D., M.R.C.P.

*Surgery:* Vice-President, F. Lace, F.R.C.S., and C. F. Walters, F.R.C.S.; Hon. Secretaries, A. de V. Blathwayt, M.R.C.S., and A. L. Fuller, F.R.C.S.

*Obstetrics:* Vice-Presidents, D. C. Rayner, F.R.C.S.; Hon. Secretary, W. H. Duncan, F.R.C.S. Ed.

*Pathology and Bacteriology:* Vice-Presidents, Rupert Waterhouse, M.D., M.R.C.P.; Hon. Secretary, James Cowan, M.B., R.A.M.C.

*Neurology and Psychological Medicine:* Vice-President, Norman Lavers, M.D.; Hon. Secretary, Ray Edridge, M.R.C.S., L.R.C.P.

*Therapeutics (including Balneology and Radiotherapy):* Vice-President, Preston King, M.D.; Hon. Secretary, Cecil Terry, M.B.

*Laryngology, Otology and Rhinology:* Hon. Secretary, H. N. Barnett, F.R.C.S. Ed.

*Diseases of Children:* Vice-President, Carey F. Coombs, M.D., F.R.C.P.; Hon. Secretary, Vincent Coates, M.C., M.D.

*Ophthalmology:* President, W. Mardon Beaumont, M.R.C.S. (Bath); Vice-President, C. H. Walker, F.R.C.S.; Hon. Secretary, R. Colley, M.B., D.O.M.S.

*Orthopædics:* President, Professor E. W. Hey Groves, M.S., F.R.C.S.; Hon. Secretary, J. S. Levis, M.C., M.B.

*Public Medicine:* Vice-President, G. F. Blackett, M.D. (Bath); Hon. Secretary, R. E. Thomas, M.D.

*Medical Sociology:* President, Charles E. S. Flemming, M.R.C.S., L.R.C.P.; Hon. Secretary, C. A. Marsh, M.D.; Hon.

Local General Secretary, Mr. W. G. Mumford, O.B.E., F.R.C.S.; Hon. Assistant Secretary, Dr. R. G. Gordon.

**University of London.**—Dr. Patrick Watson-Williams has been appointed to the "Semon Lectureship in Laryngology" in the London University. The lecture will be delivered at 5 p.m. on November 5th, 1925, at the Royal Society of Medicine, London.

EXAMINATION RESULTS.

**University of Bristol.**—Students of the University have recently passed the following examinations:—

DIPLOMA IN PUBLIC HEALTH.—*Pass:* V. W. W. Ryan. *Part II. (Completing Examination):* J. Ledingham. *Part I. only:* N. A. NcDonald Rodger, S. Sharpe.

M.B., Ch.B. (*Part II.*)—*Final Examination.—With Second Class Honours :* D. E. Crellin (Distinction in Medicine), C. I. Ham (Distinction in Surgery and Obstetrics), A. H. Lowther (Distinction in Pathology and Obstetrics), R. E. Lucas (Distinction in Medicine). *Pass :* A. F. Alford, K. F. Alford, A. S. Cox, S. J. H. Griffiths, F. H. Hollingshead, J. A. Hooker, K. F. Platt, D. C. Prowse, L. G. Robertson (Distinction in Obstetrics), H. Rogers, G. M. Terrell, M. M. Tewater. *In Medicine* (*Completing Examination*) *:* E. R. Clutterbuck. *In Surgery* (*Completing Examination*)*:* H. M. Dixon. *In Public Health* (*Completing Examination*) *:* A. H. Marshall. *In Group I. only* (*Surgery and Obstetrics*) *:* E. E. Benson, M. P. Posthuma, H. J. Satchwell, K. M. Willmore. *In Group II. only* (*Pathology, Medicine and Public Health*) *:* C. F. R. Killick.

M.B., Ch.B. (*Part I.*)—*Final Examination.—Pass* (*including Forensic Medicine and Toxicology*) *:* G. W. R. Bishop, J. F. O. Bodman, M. E. Drew, F. S. Dymond, G. L. Feneley, F. R. Gedye, A. P. Gorham, M. B. Harvey, T. P. Lalonde, M. E. J. Packer, C. B. Perry, L. B. Phillips, E. S. Rogers, A. A. B. Vincent.

M.B., Ch.B. (*Part I.*) *2nd Examination.—Pass :* I. J. Armstrong, R. A. S. Cory, A. A. Dowling, G. R. Fells, A. C. Fisher, N. D. Gerrish, A. D. James, R. J. Krogh, M. F. Potter, K. H. Pridie, H. D. Pyke, N. C. Tanner, L. E. Vine, G. J. West. *Part II. :* C. D. J. Ball, T. H. Berrill, T. A. Brand, T. L. Cleave, H. B. Murgoci (with Distinction in Anatomy), R. M. Norman, E. M. Redman, P. C. Vine, T. B. Wansbrough, A. R. Williams (with Distinction in Anatomy).

B.D.S.—*2nd Examination.—Pass* (*in Dental Anatomy only*) *:* W. M. Evans.

F.R.C.S. Eng.—*Final Examination :* J. R. Nicholson-Lailey, M.B., Ch.B. (Bristol), M.R.C.S., L.R.C.P.

M.R.C.S.—*Final Examination :* Elizabeth E. Benson, R. S. Kemm, W. George Rees Morris, Dorothy E. Crellin, Constance I. Ham, J. E. S. Hamilton, F. S. Hunter (Birmingham), A. C. Lysaght, D. C. Prowse, S. M. T. Smith.

Primary F.R.C.S. Eng.—E. Langford, M.B., Ch.B., Bristol.

L.D.S.—*Final Examination* (*Part I.*) *:* H. Hazell. *Part II.* (*Completing Examination*) *:* G. Meadows, C. I. T. Morgan.

---

Medical colleagues who are not members of the Society are invited to subscribe to the *Bristol Medico-Chirurgical Journal.* 10s. 6d. annually.

### PUBLICATIONS RECEIVED.

From E. Arnold & Co., London:

*A Manual of Gynæcology.* By S. J. Cameron, M.B., B.Ch., F.R.F.P.S.G. Third Edition. 1925. Price 25s. net.

From Cassell & Co. Ltd., London:
*Modern Operative Surgery.* Edited by H. W. Carson, F.R.C.S. Eng. 2 vols. 1924. Price £3 3s. net.
*Manson's Tropical Diseases.* Edited by Philip Manson-Bahr, D.S.O., M.A., M.D., D.T.M. & H. Cantab., F.R.C.P. Lond. Eighth Edition. 1925. Price 31s. 6d. net.

From the Delegates of the Clarendon Press, Oxford:
*The Life of Sir William Osler.* By Harvey Cushing. 2 vols. 1925. Price 37s. 6d. net for the 2 vols.

From W. Green & Son Ltd., Edinburgh:
*An Introduction to Dermatology.* By Sir Norman Walker. Eighth Edition. 1925. Price 20s. net.

From Wm. Heinemann (Medical Books) Ltd.:
*The Medical Year Book,* 1925. (Second Annual Issue.) Edited by C. R. Hewitt. Price 12s. 6d. net.
*Treatment of Gonococcal Infection by Diathermy.* By E. P. Cumberbatch, M.A., B.M., B.Ch. (Oxon)., M.R.C.P., and C. A. Robinson, M.B., B.Ch. (Cantab.), D.M.R.E. (Cantab.). 1925. Price 7s. 6d. net.
*Health and Psychology of the Child.* Edited by Elizabeth S. Chesser, M.D. 1925. Price 7s. 6d. net.
*A Textbook of Pathology, General and Special.* By J. Martin Beattie, M.A. (N.Z.), M.D. (Edin.), M.R.C.S., L.R.C.P. (Lond)., and W. E. Carnegie Dickson, M.D., B.Sc., F.R.C.P. (Edin.). Third Edition. 1925. Price 42s. net.

From H. K. Lewis & Co. Ltd., London:
*The Bacteriology of Food.* By Cuthbert Dukes, M.D., M.Sc., D.P.H. 1925. Price 3s. 6d. net.
*Essential of Infant Feeding.* By E. A. Barton. 1925. Price 3s. 6d. net.
*Minor Surgery.* By Lionel R. Fifield, F.R.C.S. (Eng.). 1925. Price 12s. 6d. net.
*An Explanation of Hydrogen Ion Concentration.* By Henry A. Ellis, B.A., M.B., Ch.B. 1925. Price 1s. net. Paper covers.

From J. B. Lippincott Company, London:
*The Crippled Hand and Arm.* By Carl Beck, M.D. 1925. Price 30s. net.

From Macmillan & Co. Ltd.:
*Medical Education.* By Abraham Flexner. 1925. Price 10s. 6d. net.

From Humphrey Milford, Oxford University Press:
*Surgical Operations.* A Textbook for Students and Nurses. By E. W. Hey Groves. Second Edition. 1925. Price 21s. net.
*Clinical Researches in Acute Abdominal Disease.* By Zachary Cope, B.A., M.D., M.S., F.R.C.S. 1925. Price 12s. 6d. net.
*Practical Obstetrics.* By E. Hastings Tweedy, M.D. Dubl., F.R.C.P.I., and G. T. Wrench, M.D., in collaboration with Bethel Solomons, M.D., F.R.C.P.I. Fifth Edition. 1925. Price 21s. net.
*Middle Age and Old Age.* By Leonard Williams, M.D.. 1925. Price 10s. 6d. net.
*Text-book for Nurses.* By E. W. Hey Groves, M.D, B.Sc., M.S., F.R.C.S., and the late J. M. Fortescue-Brickdale, M.A., M.D., M.R.C.P. The Medical Section revised by J. A. Nixon, C.M.G., M.D., F.R.C.P. Third Edition. 1925. Price 20s. net.
*Parasitology for Medical Students.* By Alex. M. Kennedy, M.D. (Glas.). 1925. Price 10s. 6d. net.

From The Scientific Press Ltd., London:

C.M.B. *Examination Questions and Model Answers.* A Handbook for Nurses. Second Edition. 1925. Price 1s. 6d. net.

*Gynæcology for Nurses and Gynæcological Nursing.* By Comyns Berkeley, M.A., M.D., M.C. Cantab., F.R.C.P., M.R.C.S. Fourth Edition. 1925. Price 7s. 6d. net.

*Medical Electricity for Nurses.* By Harold Wigg. 1925. Price 6s. net.

*The Coming of Baby.* By Lucy E. Ashby and Kate A. L. Earp. With a foreword by Sir James Cantlie, K.B.E., M.A., etc. 1925. Price 2s. net.

From J. Wright & Sons Ltd., Bristol:

*A Contribution to the Study of Pernicious Anæmia and Aplastic Anæmia.* By A. Sheard, M.D. 1924. Price 7s. net.

*The British Journal of Surgery.* January and April, 1925. Price 12s. 6d. net. Yearly subscription 42s. net.

*Tuberculosis: Its Prevention and Treatment.* By John Laird. Second Edition. 1925. Price 5s. 6d. net.

*Leprosy.* By Sir Leonard Rogers, C.I.E., M.D., F.R.C.P., F.R.C.S., F.R.S., I.M.S. (Ret.), and Ernest Muir, M.D., F.R.C.S. (Edin.). 1925. Price 12s. 6d. (cloth), 10s. 6d. (paper).

*Synopsis of Midwifery and Gynæcology.* By Aleck W. Bourne, B.A., M.B., B.Ch., F.R.C.S. Third Edition. 1925. Price 15s.

From The Year Book Publishers, Chicago:

*The Practical Medicine Series.* Vol. I. General Medicine. Edited by G. H. Weaver, M.D., and others. 1924. Price \$3. Series of eight, \$15.

